# Supplementary figures and images for: Automated Proofreading of Digitally Reconstructed Neural Morphology Enhances Accuracy, Scalability, and Standardization
Source: bioRxiv. 2026 Apr 7:2026.03.27.714818. Preprint. [Version 2] doi: 10.64898/2026.03.27.714818 (PMC13060139; doi:10.64898/2026.03.27.714818)

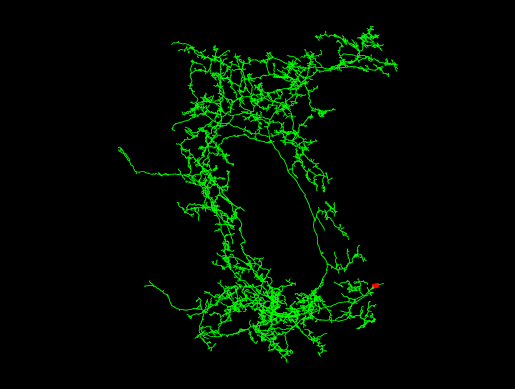

Supplement: Supplement 1 [file media-1.zip › swc_standardized_3_6_2026__23_06_30/Images/PNG/720575940616379707.png]

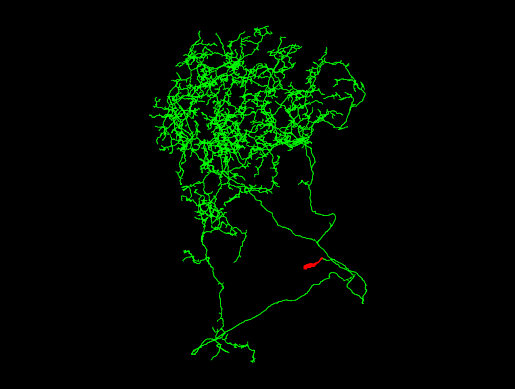

Supplement: Supplement 1 [file media-1.zip › swc_standardized_3_6_2026__23_06_30/Images/PNG/720575940616383193.png]

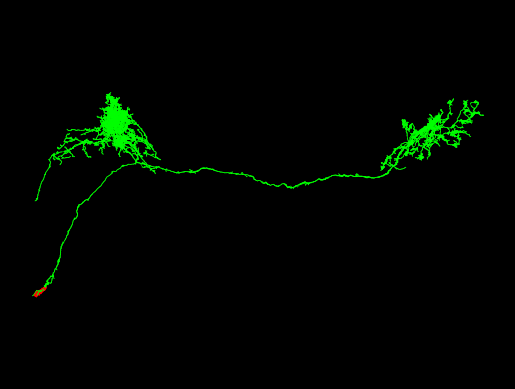

Supplement: Supplement 1 [file media-1.zip › swc_standardized_3_6_2026__23_06_30/Images/PNG/720575940616390261.png]

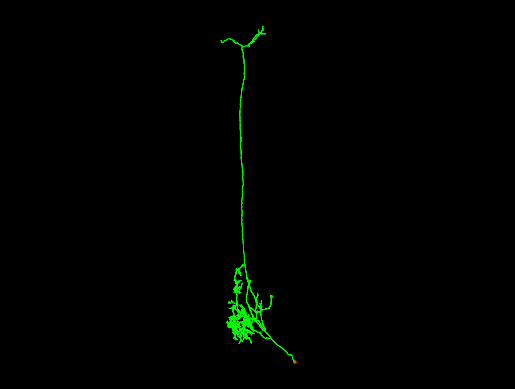

Supplement: Supplement 1 [file media-1.zip › swc_standardized_3_6_2026__23_06_30/Images/PNG/720575940616392849.png]

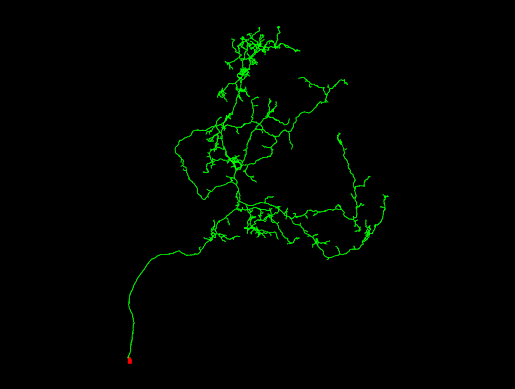

Supplement: Supplement 1 [file media-1.zip › swc_standardized_3_6_2026__23_06_30/Images/PNG/720575940616399645.png]

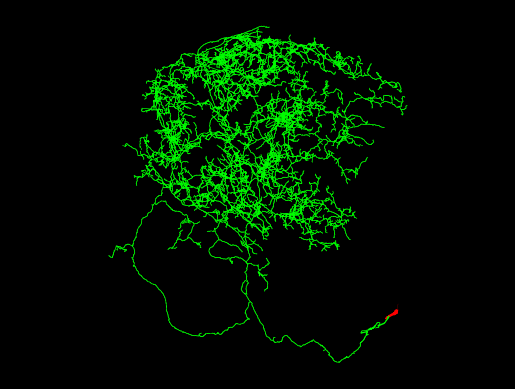

Supplement: Supplement 1 [file media-1.zip › swc_standardized_3_6_2026__23_06_30/Images/PNG/720575940616402269.png]

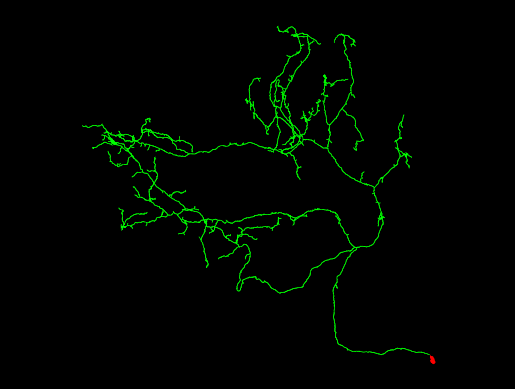

Supplement: Supplement 1 [file media-1.zip › swc_standardized_3_6_2026__23_06_30/Images/PNG/720575940616408065.png]

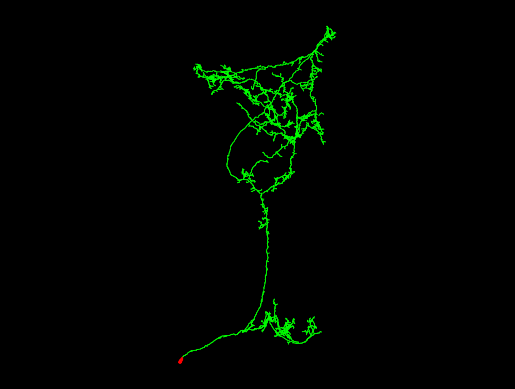

Supplement: Supplement 1 [file media-1.zip › swc_standardized_3_6_2026__23_06_30/Images/PNG/720575940616408262.png]

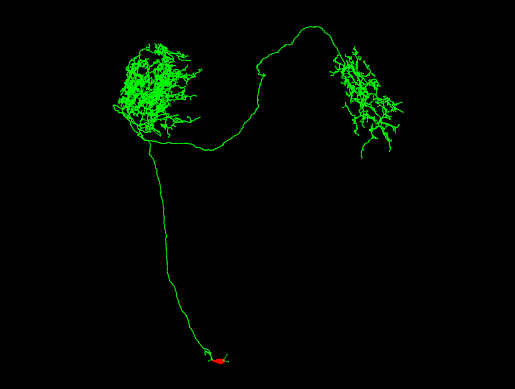

Supplement: Supplement 1 [file media-1.zip › swc_standardized_3_6_2026__23_06_30/Images/PNG/720575940616410171.png]

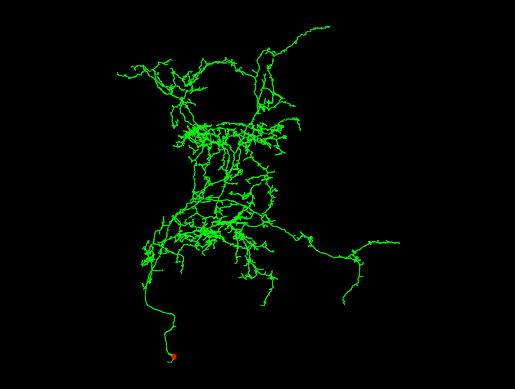

Supplement: Supplement 1 [file media-1.zip › swc_standardized_3_6_2026__23_06_30/Images/PNG/720575940616410566.png]

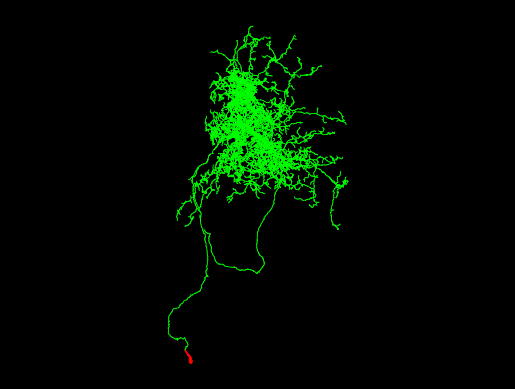

Supplement: Supplement 1 [file media-1.zip › swc_standardized_3_6_2026__23_06_30/Images/PNG/720575940616413894.png]

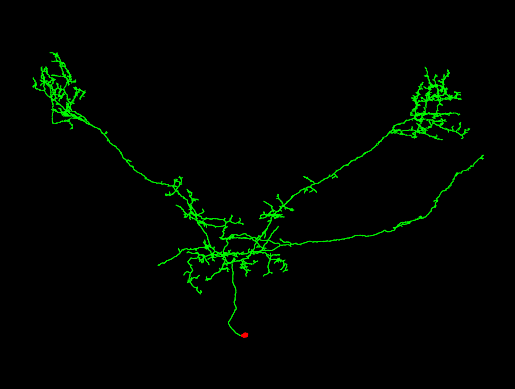

Supplement: Supplement 1 [file media-1.zip › swc_standardized_3_6_2026__23_06_30/Images/PNG/720575940616415003.png]

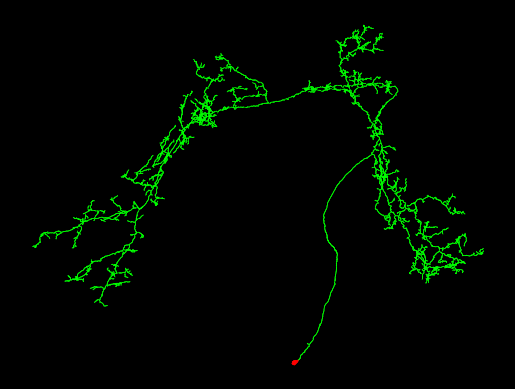

Supplement: Supplement 1 [file media-1.zip › swc_standardized_3_6_2026__23_06_30/Images/PNG/720575940616420865.png]

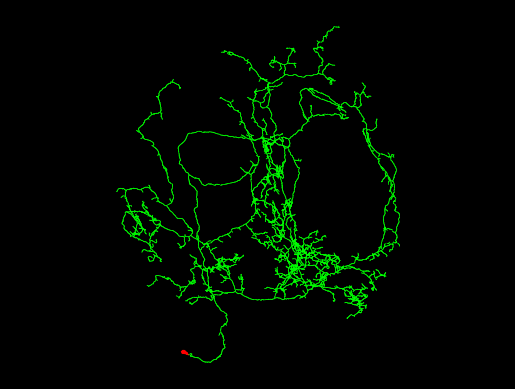

Supplement: Supplement 1 [file media-1.zip › swc_standardized_3_6_2026__23_06_30/Images/PNG/720575940616425531.png]

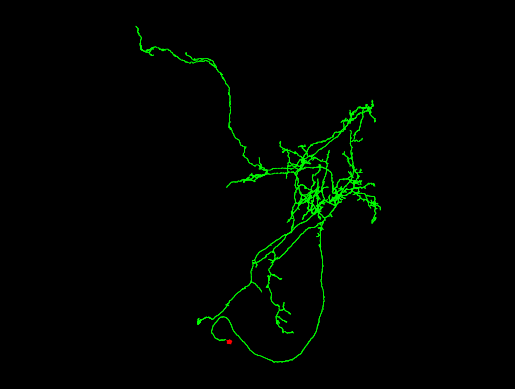

Supplement: Supplement 1 [file media-1.zip › swc_standardized_3_6_2026__23_06_30/Images/PNG/720575940616429825.png]

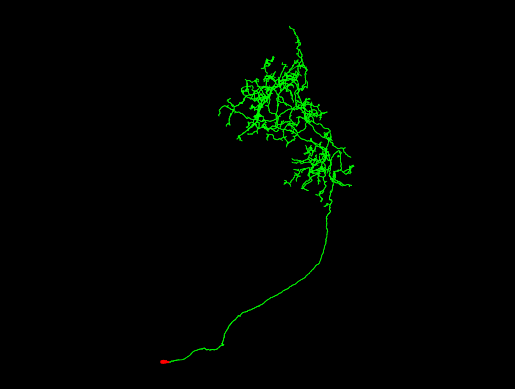

Supplement: Supplement 1 [file media-1.zip › swc_standardized_3_6_2026__23_06_30/Images/PNG/720575940616441181.png]

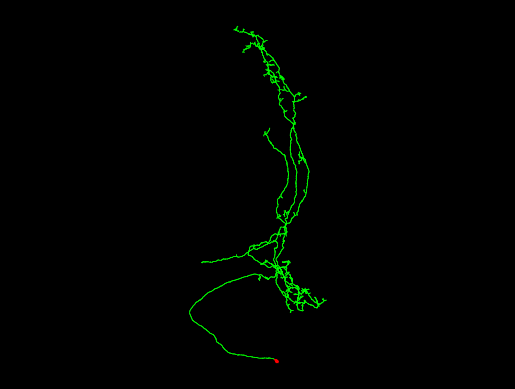

Supplement: Supplement 1 [file media-1.zip › swc_standardized_3_6_2026__23_06_30/Images/PNG/720575940616448581.png]

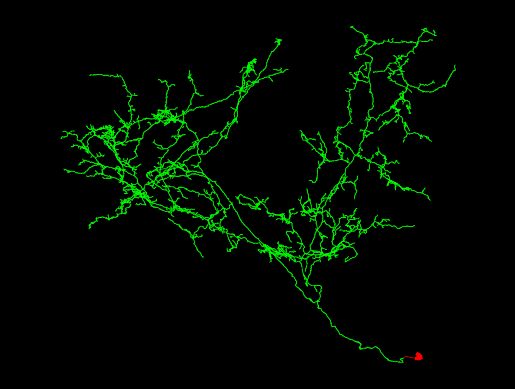

Supplement: Supplement 1 [file media-1.zip › swc_standardized_3_6_2026__23_06_30/Images/PNG/720575940616451909.png]

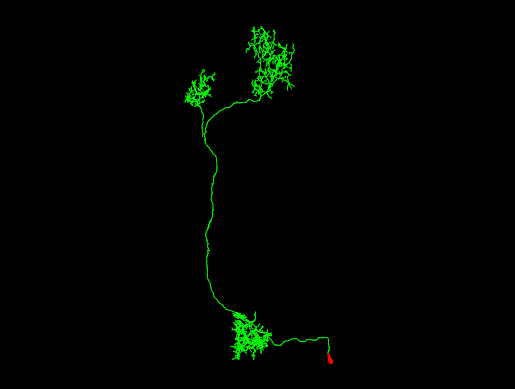

Supplement: Supplement 1 [file media-1.zip › swc_standardized_3_6_2026__23_06_30/Images/PNG/720575940616459333.png]

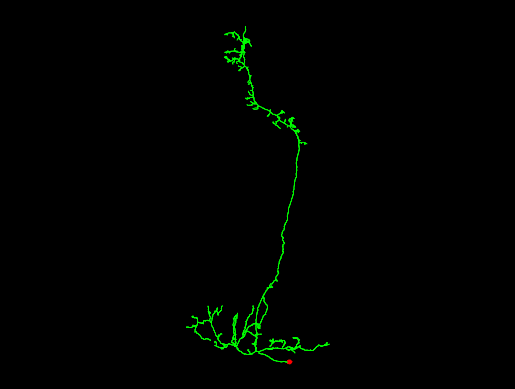

Supplement: Supplement 1 [file media-1.zip › swc_standardized_3_6_2026__23_06_30/Images/PNG/720575940616462987.png]

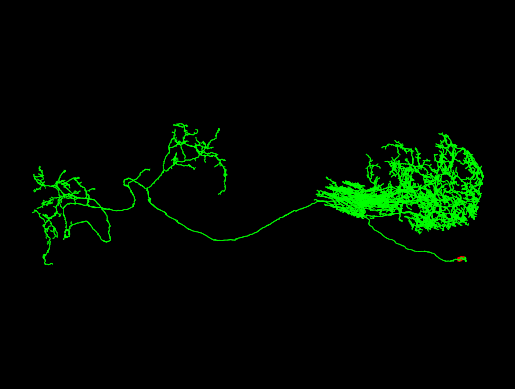

Supplement: Supplement 1 [file media-1.zip › swc_standardized_3_6_2026__23_06_30/Images/PNG/720575940616463477.png]

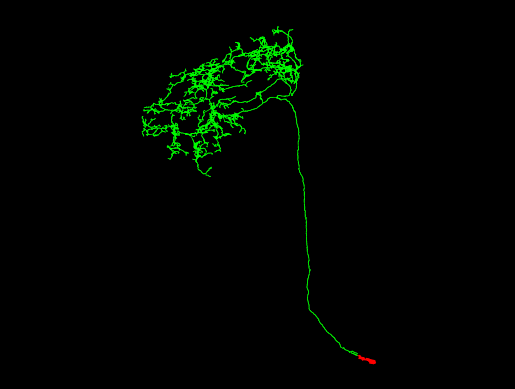

Supplement: Supplement 1 [file media-1.zip › swc_standardized_3_6_2026__23_06_30/Images/PNG/720575940616464340.png]

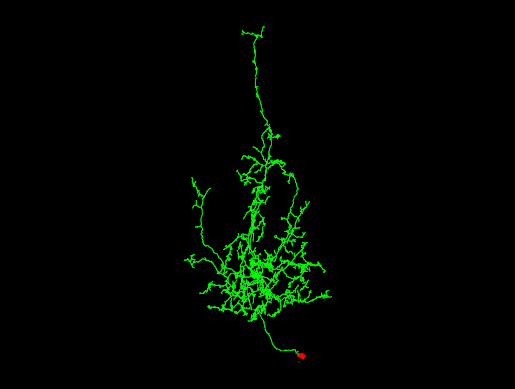

Supplement: Supplement 1 [file media-1.zip › swc_standardized_3_6_2026__23_06_30/Images/PNG/720575940616466260.png]

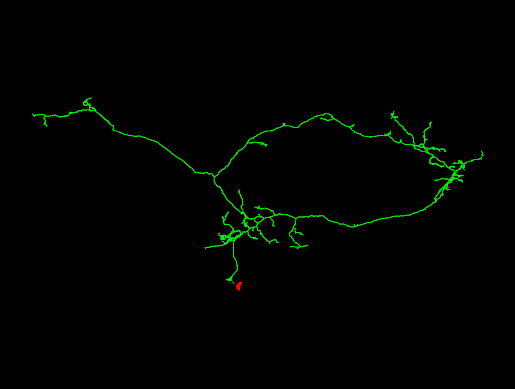

Supplement: Supplement 1 [file media-1.zip › swc_standardized_3_6_2026__23_06_30/Images/PNG/720575940616466388.png]

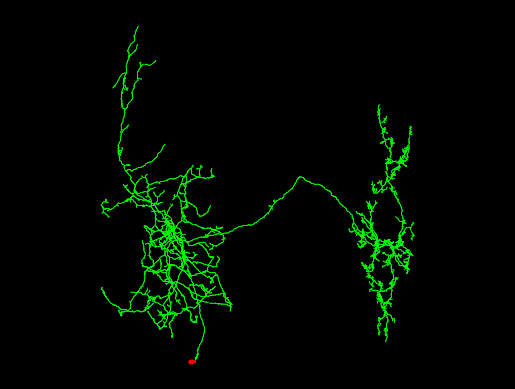

Supplement: Supplement 1 [file media-1.zip › swc_standardized_3_6_2026__23_06_30/Images/PNG/720575940616466805.png]

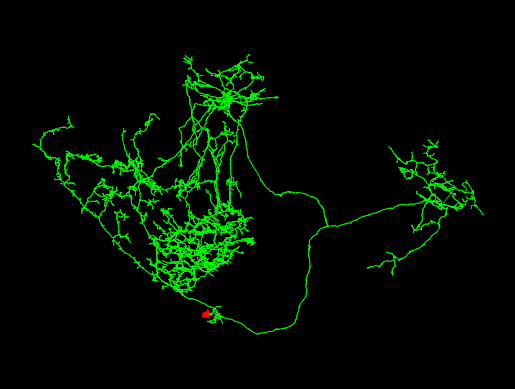

Supplement: Supplement 1 [file media-1.zip › swc_standardized_3_6_2026__23_06_30/Images/PNG/720575940616481397.png]

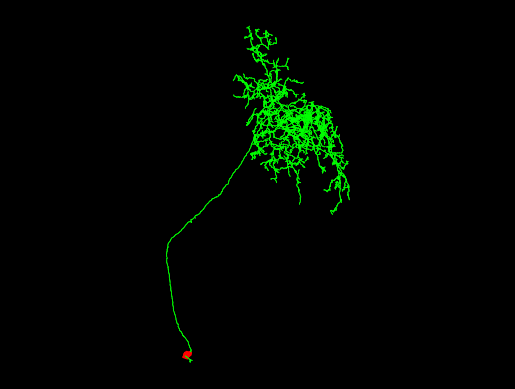

Supplement: Supplement 1 [file media-1.zip › swc_standardized_3_6_2026__23_06_30/Images/PNG/720575940616483284.png]

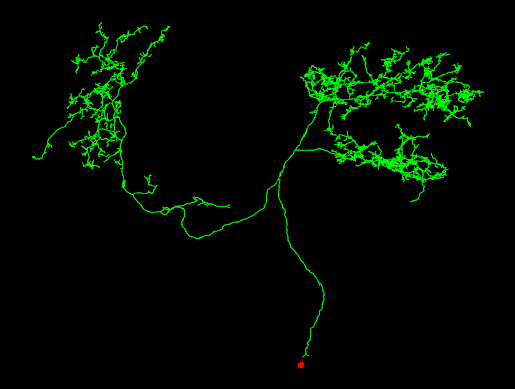

Supplement: Supplement 1 [file media-1.zip › swc_standardized_3_6_2026__23_06_30/Images/PNG/720575940616483414.png]

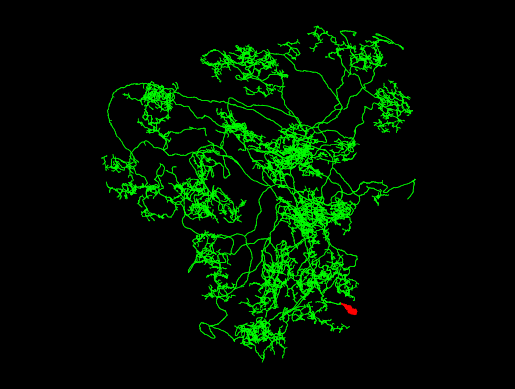

Supplement: Supplement 1 [file media-1.zip › swc_standardized_3_6_2026__23_06_30/Images/PNG/720575940616486230.png]

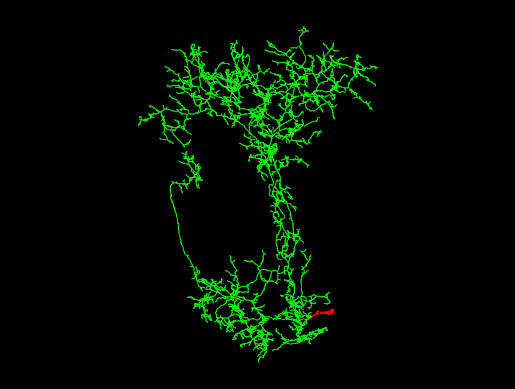

Supplement: Supplement 1 [file media-1.zip › swc_standardized_3_6_2026__23_06_30/Images/PNG/720575940616501877.png]

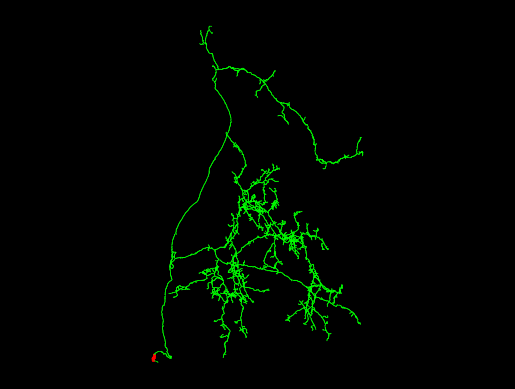

Supplement: Supplement 1 [file media-1.zip › swc_standardized_3_6_2026__23_06_30/Images/PNG/720575940616502109.png]

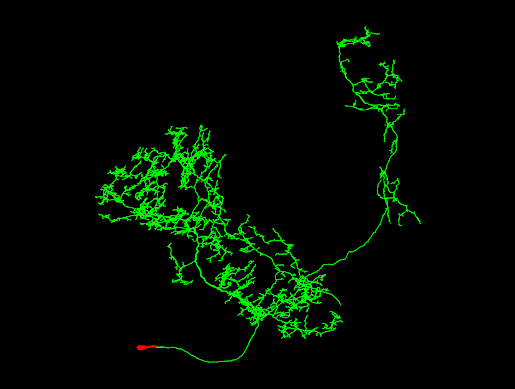

Supplement: Supplement 1 [file media-1.zip › swc_standardized_3_6_2026__23_06_30/Images/PNG/720575940616504977.png]

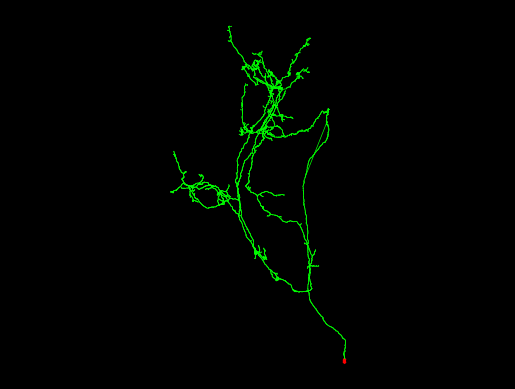

Supplement: Supplement 1 [file media-1.zip › swc_standardized_3_6_2026__23_06_30/Images/PNG/720575940616505017.png]

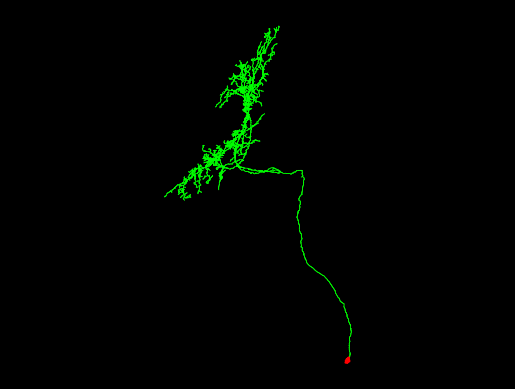

Supplement: Supplement 1 [file media-1.zip › swc_standardized_3_6_2026__23_06_30/Images/PNG/720575940616507865.png]

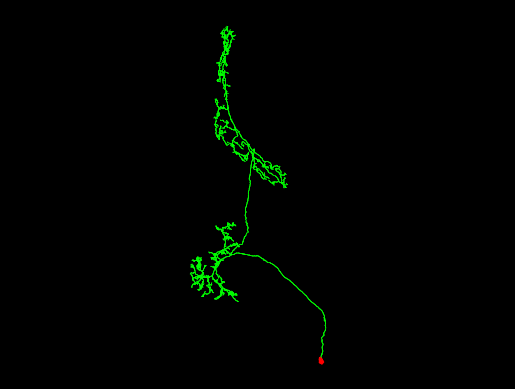

Supplement: Supplement 1 [file media-1.zip › swc_standardized_3_6_2026__23_06_30/Images/PNG/720575940616511813.png]

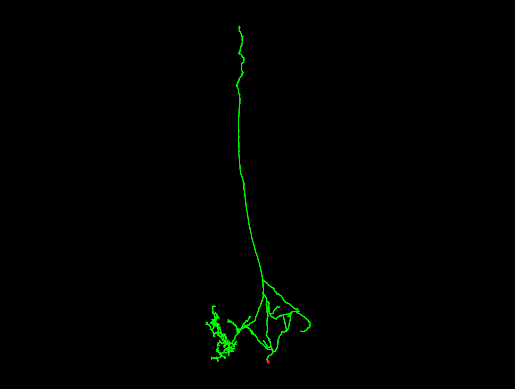

Supplement: Supplement 1 [file media-1.zip › swc_standardized_3_6_2026__23_06_30/Images/PNG/720575940616525429.png]

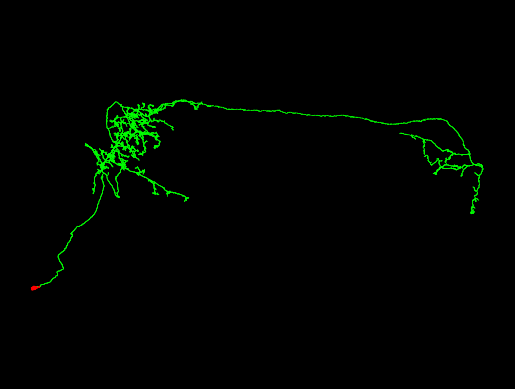

Supplement: Supplement 1 [file media-1.zip › swc_standardized_3_6_2026__23_06_30/Images/PNG/720575940616538944.png]

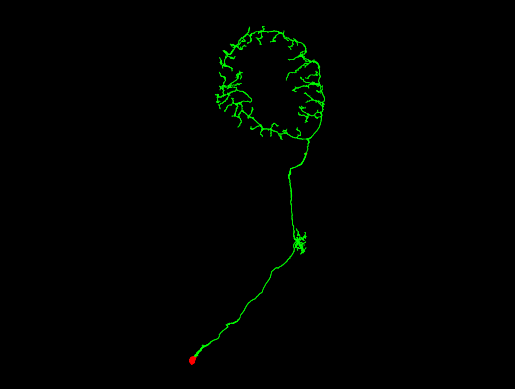

Supplement: Supplement 1 [file media-1.zip › swc_standardized_3_6_2026__23_06_30/Images/PNG/720575940616539990.png]

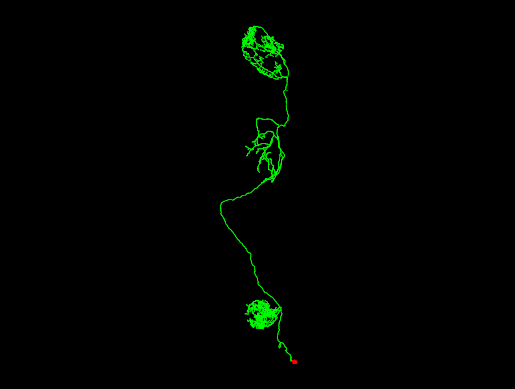

Supplement: Supplement 1 [file media-1.zip › swc_standardized_3_6_2026__23_06_30/Images/PNG/720575940616543745.png]

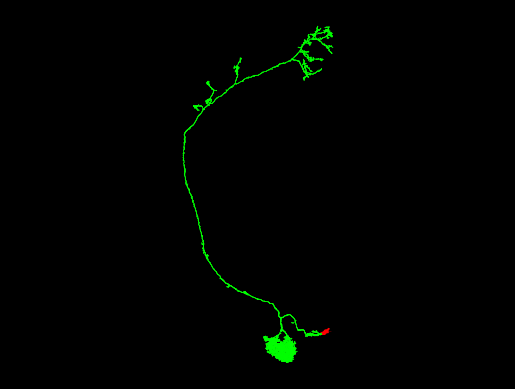

Supplement: Supplement 1 [file media-1.zip › swc_standardized_3_6_2026__23_06_30/Images/PNG/720575940616566133.png]

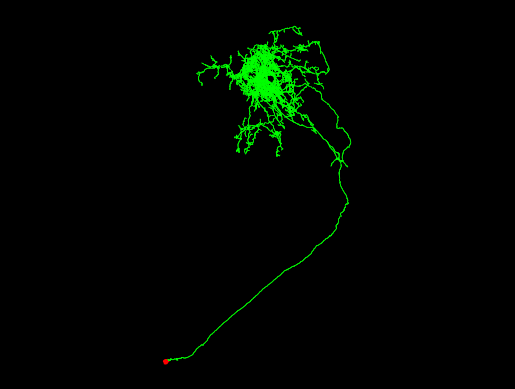

Supplement: Supplement 1 [file media-1.zip › swc_standardized_3_6_2026__23_06_30/Images/PNG/720575940616702465.png]

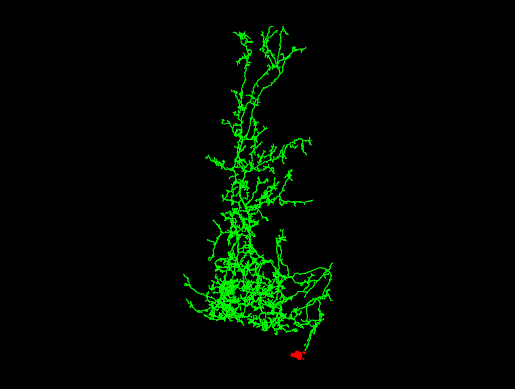

Supplement: Supplement 1 [file media-1.zip › swc_standardized_3_6_2026__23_06_30/Images/PNG/720575940616848214.png]

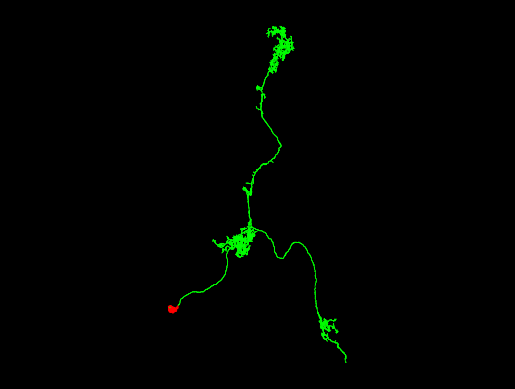

Supplement: Supplement 1 [file media-1.zip › swc_standardized_3_6_2026__23_06_30/Images/PNG/720575940617422641.png]

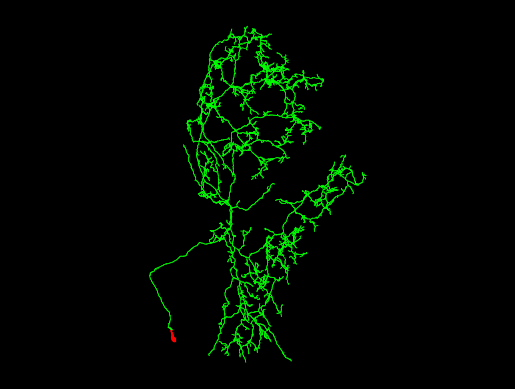

Supplement: Supplement 1 [file media-1.zip › swc_standardized_3_6_2026__23_06_30/Images/PNG/720575940617425593.png]

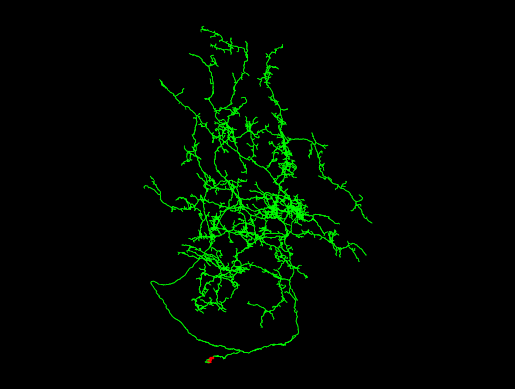

Supplement: Supplement 1 [file media-1.zip › swc_standardized_3_6_2026__23_06_30/Images/PNG/720575940617429853.png]

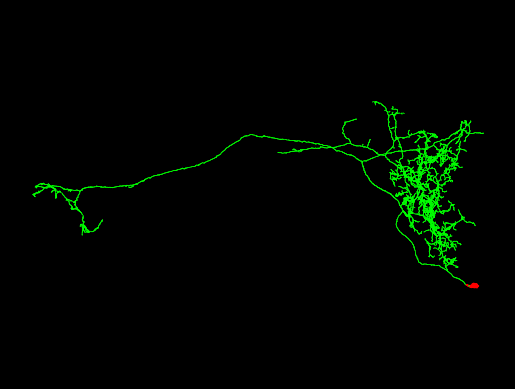

Supplement: Supplement 1 [file media-1.zip › swc_standardized_3_6_2026__23_06_30/Images/PNG/720575940617431325.png]

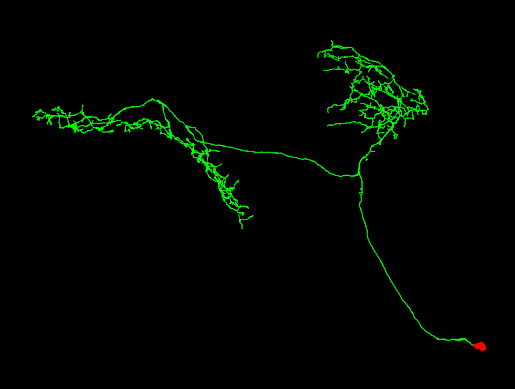

Supplement: Supplement 1 [file media-1.zip › swc_standardized_3_6_2026__23_06_30/Images/PNG/720575940617433627.png]

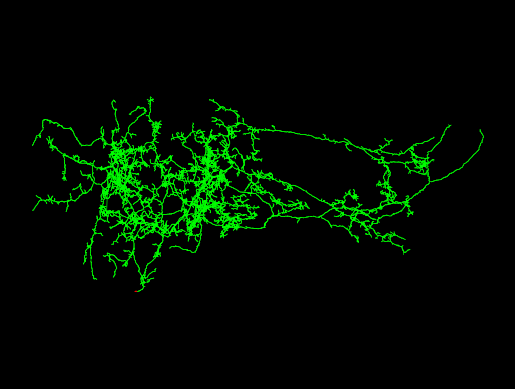

Supplement: Supplement 1 [file media-1.zip › swc_standardized_3_6_2026__23_06_30/Images/PNG/720575940617440395.png]

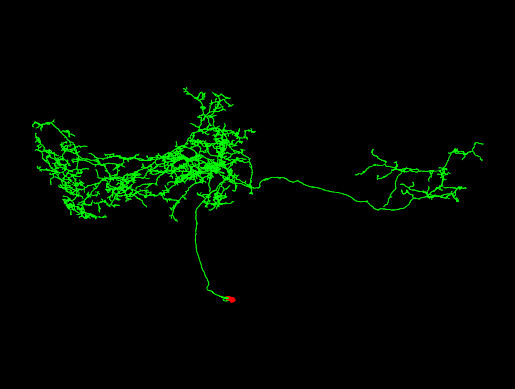

Supplement: Supplement 1 [file media-1.zip › swc_standardized_3_6_2026__23_06_30/Images/PNG/720575940617440802.png]

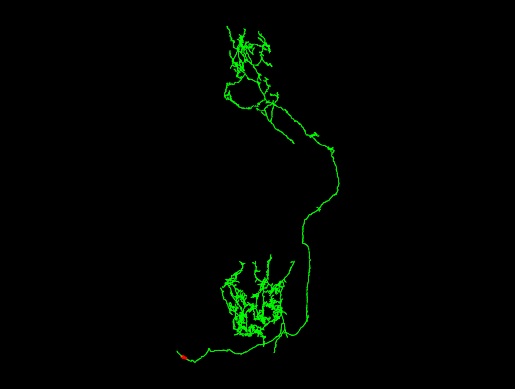

Supplement: Supplement 1 [file media-1.zip › swc_standardized_3_6_2026__23_06_30/Images/PNG/720575940617441752.png]

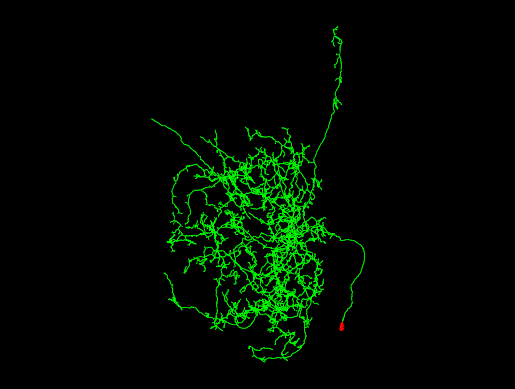

Supplement: Supplement 1 [file media-1.zip › swc_standardized_3_6_2026__23_06_30/Images/PNG/720575940617452500.png]

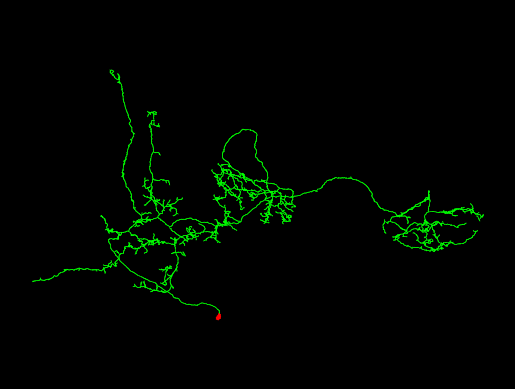

Supplement: Supplement 1 [file media-1.zip › swc_standardized_3_6_2026__23_06_30/Images/PNG/720575940617466395.png]

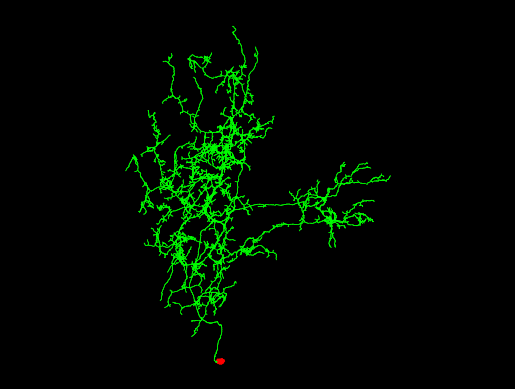

Supplement: Supplement 1 [file media-1.zip › swc_standardized_3_6_2026__23_06_30/Images/PNG/720575940617467712.png]

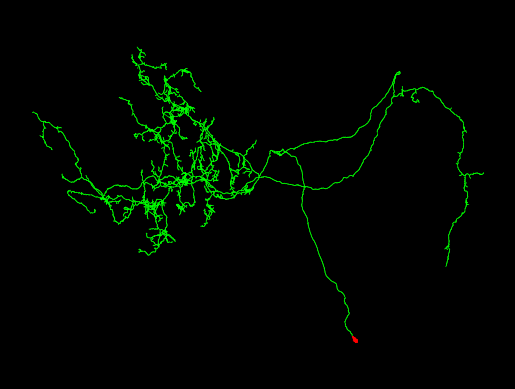

Supplement: Supplement 1 [file media-1.zip › swc_standardized_3_6_2026__23_06_30/Images/PNG/720575940617470164.png]

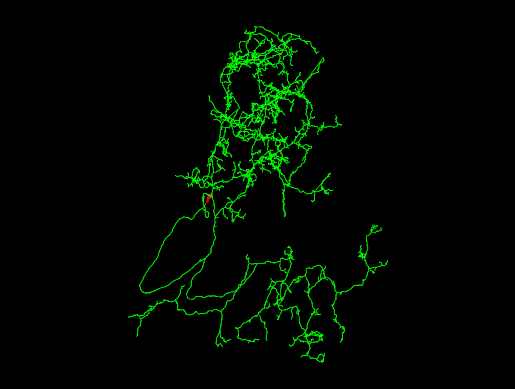

Supplement: Supplement 1 [file media-1.zip › swc_standardized_3_6_2026__23_06_30/Images/PNG/720575940617470932.png]

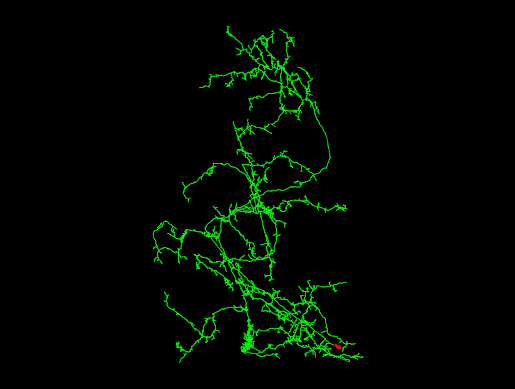

Supplement: Supplement 1 [file media-1.zip › swc_standardized_3_6_2026__23_06_30/Images/PNG/720575940617475028.png]

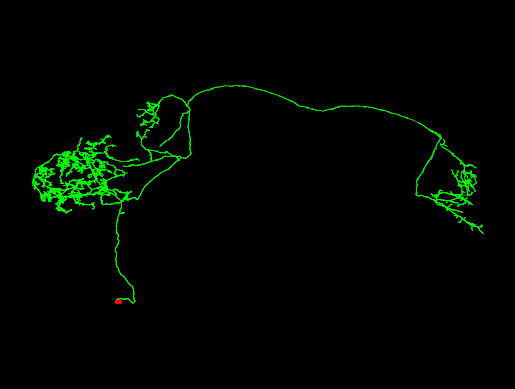

Supplement: Supplement 1 [file media-1.zip › swc_standardized_3_6_2026__23_06_30/Images/PNG/720575940617476052.png]

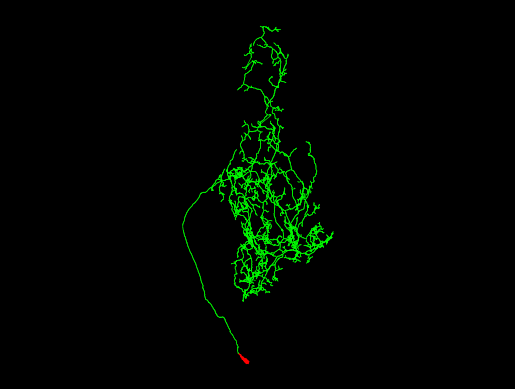

Supplement: Supplement 1 [file media-1.zip › swc_standardized_3_6_2026__23_06_30/Images/PNG/720575940617476379.png]

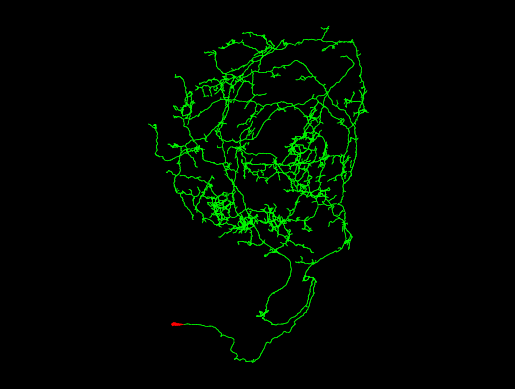

Supplement: Supplement 1 [file media-1.zip › swc_standardized_3_6_2026__23_06_30/Images/PNG/720575940617479202.png]

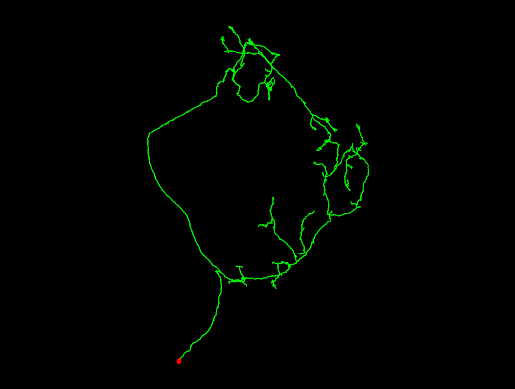

Supplement: Supplement 1 [file media-1.zip › swc_standardized_3_6_2026__23_06_30/Images/PNG/720575940617479707.png]

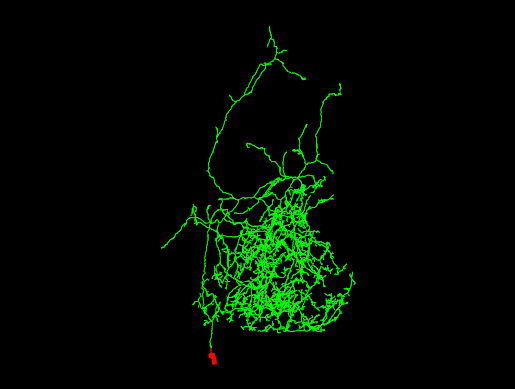

Supplement: Supplement 1 [file media-1.zip › swc_standardized_3_6_2026__23_06_30/Images/PNG/720575940617488477.png]

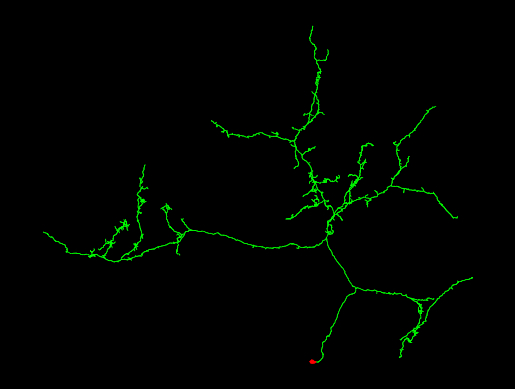

Supplement: Supplement 1 [file media-1.zip › swc_standardized_3_6_2026__23_06_30/Images/PNG/720575940617489409.png]

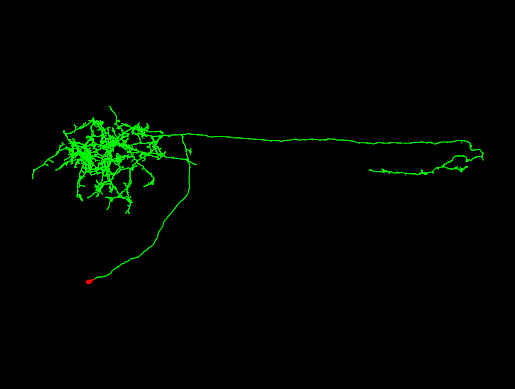

Supplement: Supplement 1 [file media-1.zip › swc_standardized_3_6_2026__23_06_30/Images/PNG/720575940617492821.png]

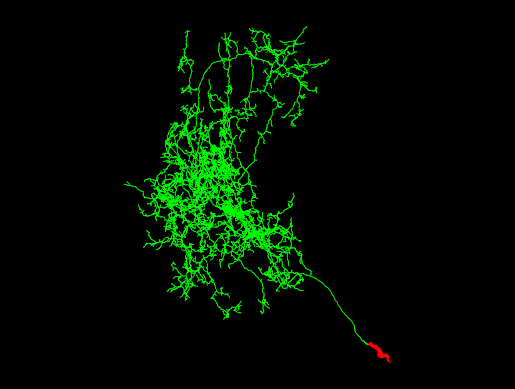

Supplement: Supplement 1 [file media-1.zip › swc_standardized_3_6_2026__23_06_30/Images/PNG/720575940617493460.png]

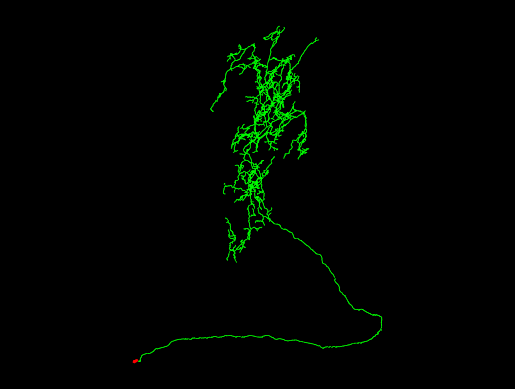

Supplement: Supplement 1 [file media-1.zip › swc_standardized_3_6_2026__23_06_30/Images/PNG/720575940617494417.png]

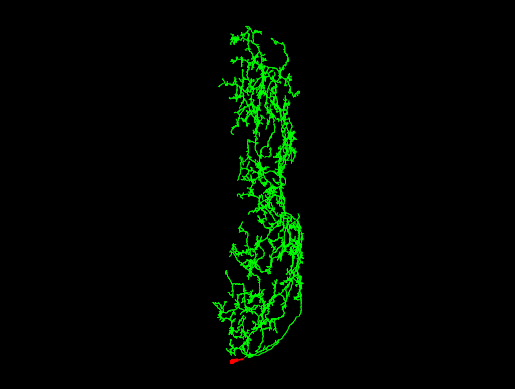

Supplement: Supplement 1 [file media-1.zip › swc_standardized_3_6_2026__23_06_30/Images/PNG/720575940617494877.png]

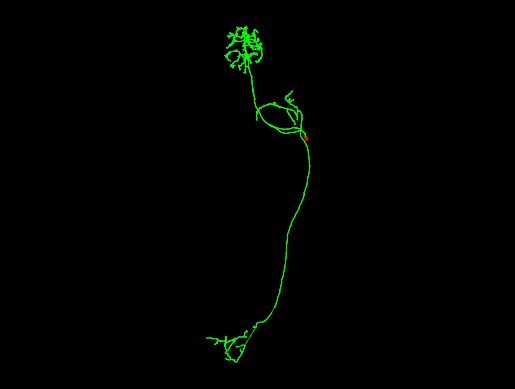

Supplement: Supplement 1 [file media-1.zip › swc_standardized_3_6_2026__23_06_30/Images/PNG/720575940617496209.png]

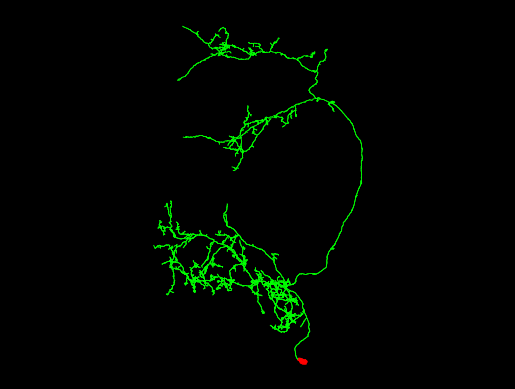

Supplement: Supplement 1 [file media-1.zip › swc_standardized_3_6_2026__23_06_30/Images/PNG/720575940617496861.png]

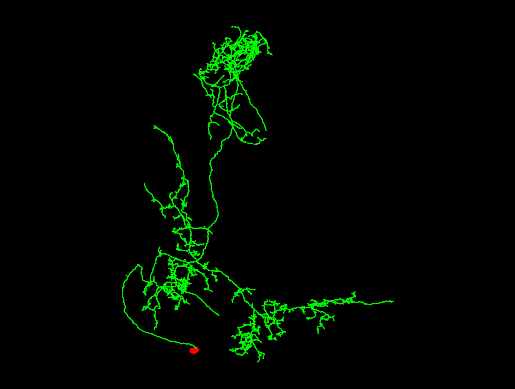

Supplement: Supplement 1 [file media-1.zip › swc_standardized_3_6_2026__23_06_30/Images/PNG/720575940617496925.png]

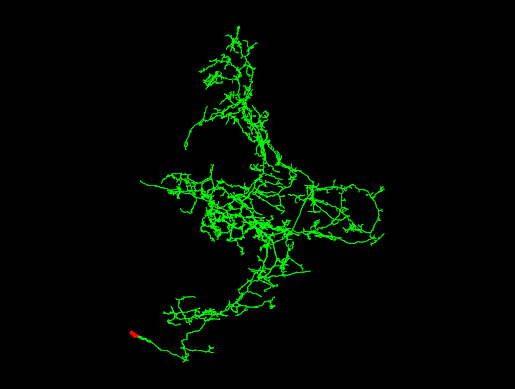

Supplement: Supplement 1 [file media-1.zip › swc_standardized_3_6_2026__23_06_30/Images/PNG/720575940617497437.png]

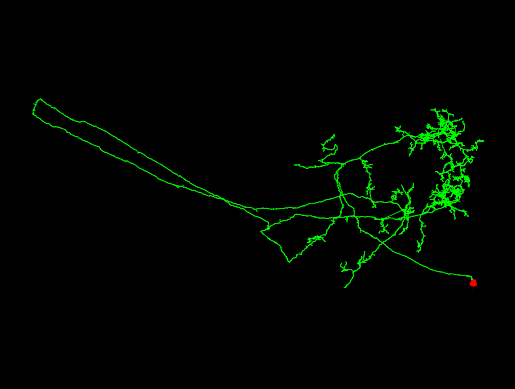

Supplement: Supplement 1 [file media-1.zip › swc_standardized_3_6_2026__23_06_30/Images/PNG/720575940617498717.png]

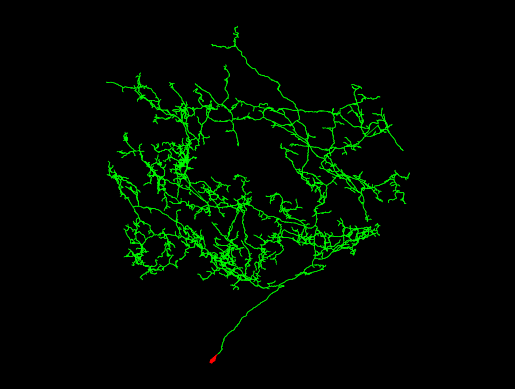

Supplement: Supplement 1 [file media-1.zip › swc_standardized_3_6_2026__23_06_30/Images/PNG/720575940617499205.png]

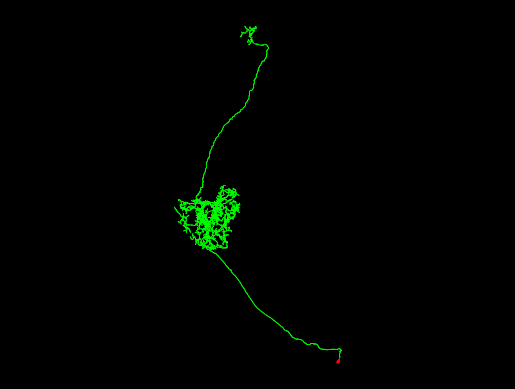

Supplement: Supplement 1 [file media-1.zip › swc_standardized_3_6_2026__23_06_30/Images/PNG/720575940617501533.png]

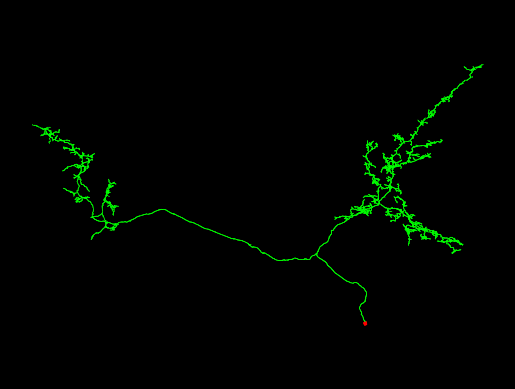

Supplement: Supplement 1 [file media-1.zip › swc_standardized_3_6_2026__23_06_30/Images/PNG/720575940617503837.png]

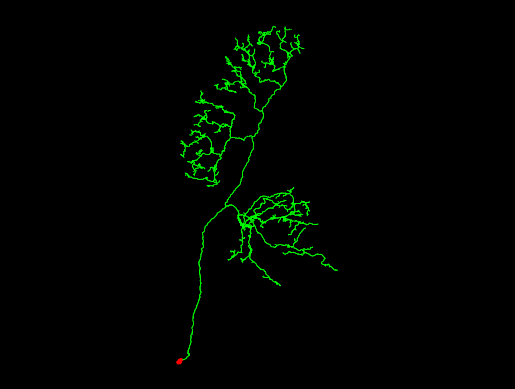

Supplement: Supplement 1 [file media-1.zip › swc_standardized_3_6_2026__23_06_30/Images/PNG/720575940618374558.png]

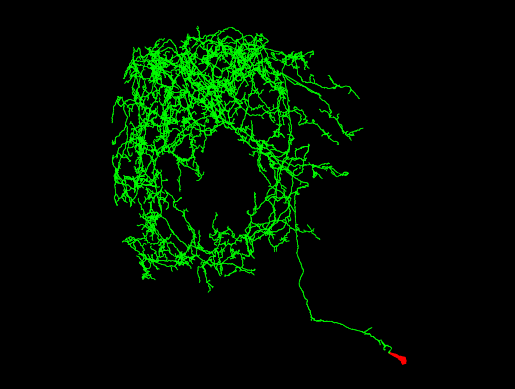

Supplement: Supplement 1 [file media-1.zip › swc_standardized_3_6_2026__23_06_30/Images/PNG/720575940618380667.png]

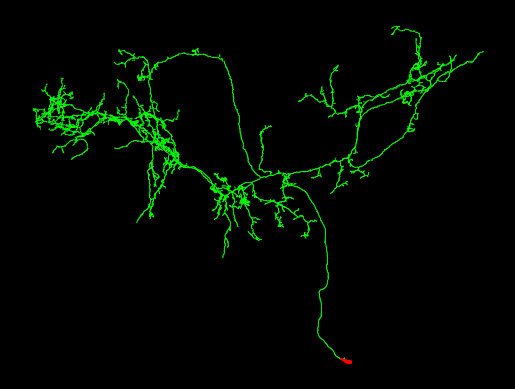

Supplement: Supplement 1 [file media-1.zip › swc_standardized_3_6_2026__23_06_30/Images/PNG/720575940618516894.png]

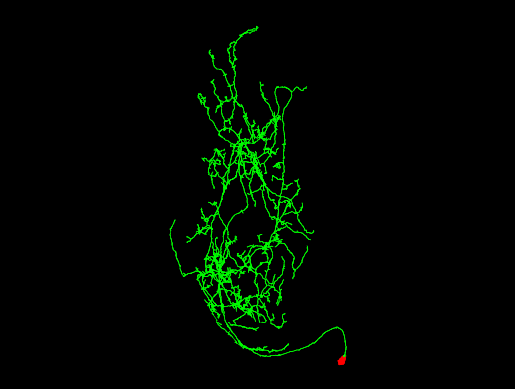

Supplement: Supplement 1 [file media-1.zip › swc_standardized_3_6_2026__23_06_30/Images/PNG/720575940618518582.png]

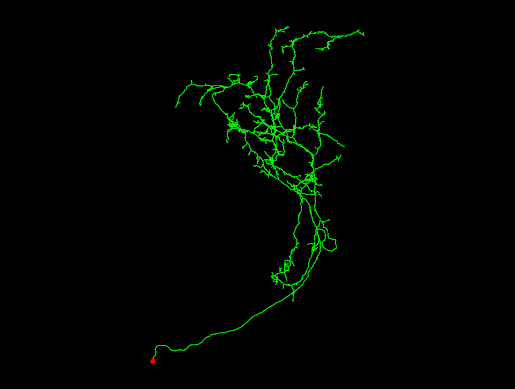

Supplement: Supplement 1 [file media-1.zip › swc_standardized_3_6_2026__23_06_30/Images/PNG/720575940618522673.png]

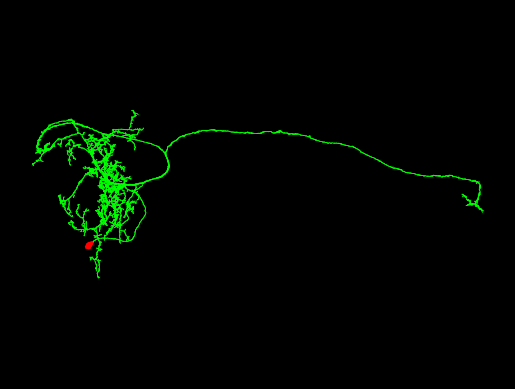

Supplement: Supplement 1 [file media-1.zip › swc_standardized_3_6_2026__23_06_30/Images/PNG/720575940618524481.png]

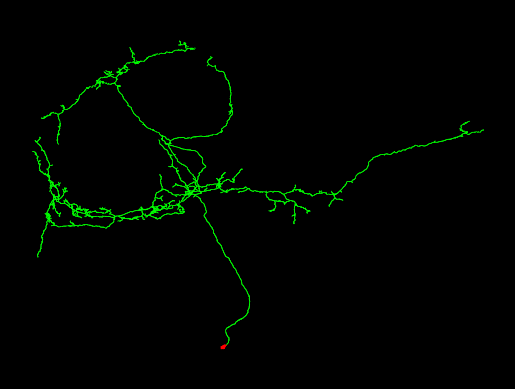

Supplement: Supplement 1 [file media-1.zip › swc_standardized_3_6_2026__23_06_30/Images/PNG/720575940618532779.png]

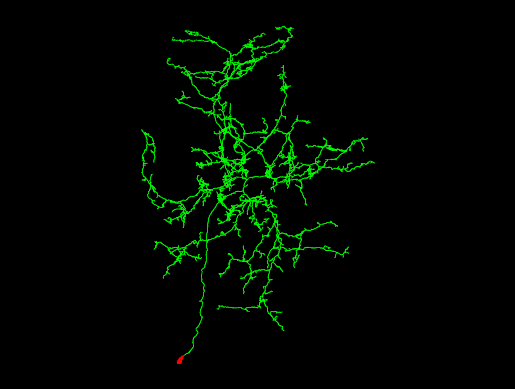

Supplement: Supplement 1 [file media-1.zip › swc_standardized_3_6_2026__23_06_30/Images/PNG/720575940618536528.png]
