# Supplementary material for: Automated Proofreading of Digitally Reconstructed Neural Morphology Enhances Accuracy, Scalability, and Standardization
Source: bioRxiv. 2026 Apr 7:2026.03.27.714818. Preprint. [Version 2] doi: 10.64898/2026.03.27.714818 (PMC13060139; doi:10.64898/2026.03.27.714818)
Supplement: Supplement 1 [file media-1.zip › AccuracyResults_20500neurons/AccuracyPerEpoch_20500neurons.docx]

Highest Eval Accuracy: 99.51%

Test Accuracy: 99.51%

Highest Eval Accuracy: 99.51%

Test Accuracy: 99.51%

Highest Eval Accuracy: 99.51%

Test Accuracy: 99.51%

Highest Eval Accuracy: 99.51%

Test Accuracy: 99.51%

Highest Eval Accuracy: 99.51%

Test Accuracy: 99.51%

Highest Eval Accuracy: 99.51%

Test Accuracy: 99.51%

Highest Eval Accuracy: 99.51%

Test Accuracy: 99.51%

Highest Eval Accuracy: 99.51%

Test Accuracy: 99.51%

Highest Eval Accuracy: 99.51%

Test Accuracy: 99.51%

Highest Eval Accuracy: 99.51%

Test Accuracy: 99.51%

Avg Test Accuracy: 99.51%

Total Elapsed Time: 1 day, 1:26:56 (Hr:Mn:SS)
